# Preliminary Comparison of Fractional Absorption of Zinc Sulphate, Zinc Gluconate, and Zinc Aspartate after Oral Supple-Mentation in Healthy Human Volunteers

**DOI:** 10.3390/nu15081885

**Published:** 2023-04-13

**Authors:** Francesco Piacenza, Robertina Giacconi, Laura Costarelli, Marco Malavolta

**Affiliations:** National Institute of Research and Care of Aging IRCCS INRCA, 60124 Ancona AN, Italy

**Keywords:** zinc, absorption, stable isotopes, zinc-aspartate, zinc-gluconate, zinc sulphate, double-blind three-period crossover trial

## Abstract

(1) Background: Zinc is generally used as a nutritional supplement for individuals at nutritional risk, such as older adults. This preliminary study investigated the fractional Zn absorption (FZA) after the supplementation on eight healthy volunteers with three different Zn complexes acquired with milk. (2) Methods: The design was a double-blind, three-period crossover trial. The volunteers were randomly divided into three groups. Each individual consumed 200 mL of bovine milk and rotated through a simultaneous administration of a single oral dose of ^70^ZnSO_4_, ^70^Zn-Gluconate (^70^Zn-Glu), and ^70^Zn-Aspartate (^70^Zn-Asp), equivalent to 2.0 mg ^70^Zn, followed by 2 weeks of wash-out. An estimation of the FZA for comparative purposes was computed by the isotopic ratio between ^66^Zn and ^70^Zn in urine collected before and 48 h after administration. (3) Results: The estimated FZA was found to be significantly higher for ^70^Zn-Asp when compared to the other forms, while the FZA of ^70^Zn-Glu was found to be significantly higher than ^70^ZnSO_4_. (4) Conclusions: The results of this study suggest that complexing Zn with aspartate in milk could be a useful tool to improve FZA in individuals at risk of Zn deficiency. These results provide a rationale for conducting further studies on Zn-Asp preparations.

## 1. Introduction

Zinc (Zn) is a trace element that plays a crucial role in various physiological processes for human health. Over 300 enzymes in the body require Zn for proper functioning, making it a vital component in most biochemical pathways. Zn is essential for immune cell development and function, wound healing, and DNA synthesis [1,2,3]. Oysters, red meat, poultry, beans, nuts, and whole grains are rich sources of Zn. Despite the worldwide availability of these foods, Zn deficiency is widespread, especially in developing countries, suggesting that malnutrition or other biological or physiological issues, not strictly related to the country’s income, contribute to this phenomenon [4]. While anyone can experience Zn deficiency, certain groups are more susceptible. These groups include: (a) vegetarians and vegans, as they lack animal Zn-rich foods in their diets; (b) pregnant and lactating women, who require more Zn to support fetal growth and milk production; (c) infants and young children, who have high Zn requirements for growth and development; (d) elderly individuals, whose ability to absorb Zn is lower; (e) individuals with gastrointestinal disorders, such as Crohn’s disease, ulcerative colitis, and celiac disease, where Zn absorption may be compromised, leading to Zn deficiency; (f) alcoholics, whose chronic alcohol consumption can impair Zn absorption and increase urinary Zn excretion, leading to Zn deficiency; (g) individuals with Sickle Cell Anemia, who require Zn for new red blood cell production; and (h)individuals with Chronic Kidney Disease, who experience increased urinary Zn excretion and decreased intestinal absorption.

Therefore, maintaining adequate Zn intake is crucial for optimal health. Though the recommended daily intake (RDI) of Zn varies depending on age and gender, the general guideline for adults is around 8–11 mg/day [5]. However, excessive Zn intake can be harmful, leading to adverse effects, such as nausea, vomiting, and diarrhea [6]. Thus, it is essential to follow the RDI guidelines and consult with a healthcare professional before starting any Zn supplementation regimen.

Interest in human Zn nutrition has increased due to the growing consumption of unrefined cereal grains, which contain high levels of phytic acid. Phytic acid is a potent inhibitor of Zn absorption [7]. Furthermore, evidence suggests that dietary Zn intake, particularly among older adults and those at risk of Zn deficiency (as previously mentioned), can often fall below the RDI.

To address Zn deficiency, a variety of Zn supplements and Zn-enriched foods, including milk, have been developed in recent years and are now available on the market. Zn supplementation has been demonstrated to effectively treat Zn deficiency and improve various health outcomes. For instance, supplementing with Zn has been found to reduce the duration and severity of cold symptoms, enhance wound healing, and improve immune function [1,2,3]. Additionally, Zn has antioxidant properties that may help protect against oxidative damage and prevent chronic diseases, such as cancer and cardiovascular disease [8]. In the elderly population, Zn supplementation has been shown to: (i) reduce the incidence of infections [9]; and (ii) improve Zn status by restoring immune function [10].

The bioavailability and functional outcomes of Zn can be influenced by the food matrix or the form of Zn used in the supplement [11,12,13,14]. Zn in supplemental forms must be absorbed across the intestine and taken up into the systemic circulation. Zn sulfate (ZnSO_4_) has been utilized as the reference compound for Zn supplementation due to its bioavailability and affordability [15,16].

Water-soluble Zn salts, including gluconate, sulfate, and acetate, are frequently utilized as supplements in tablet or syrup form to prevent Zn deficiency and treat diarrhea in children in conjunction with oral rehydration [6,17,18,19].

Zn sulfate administration in diabetic patients resulted in a significant reduction in triglyceride, low-density lipoprotein–cholesterol levels, and systolic blood pressure [20]. After 12 weeks, there was also a significant decrease in HbA1C [20]. Additionally, Zn sulfate was found to improve premenstrual syndrome and health-related quality of life in young women [21]. In an older population, supplementation with 25 mg of Zn sulfate per day enhanced cell-mediated immune response [22].

Oral ingestion of Zn acetate has been shown to reduce recurrence of respiratory and diarrheal diseases [17,18]. It mitigates the symptoms of acute dehydrating diarrheal diatheses [19], leading to recommendations of adjunctive supplementation for both developing and developed country settings.

There are concerns regarding reduced Zn absorption when given with milk or in the presence of high calcium [23,24,25,26,27,28], emphasizing the need to determine the efficiency of different forms of Zn supplements when administered with milk. Zn complexes with sugars or amino acids, such as Zn-gluconate (Zn-Glu) and Zn-aspartate (Zn-Asp), are claimed to offer better isotopic absorption than ZnSO_4_ [7].

Previous studies have shown that Zn-Glu intervention in patients with ulcerative colitis improves their nutritional status and positively influences their clinical outcome, emphasizing the importance of Zn as a dietary component in disease control [29]. In hemodialysis patients, daily consumption of Zn-Glu (30 mg) for 60 days has beneficial effects on glycemic status and lipid profile [30].

Supplementation with Zn-Asp (48 ± 2 days) at a dose of 10 mg/day as Zn aspartate resulted in a general increase in Zn levels in the supplemented sample, particularly in those with IL-6-174 polymorphism [31]. Zn-Asp also showed allogenic suppression properties in an ex vivo model, suggesting its powerful clinical application as an immunosuppressive agent [32].

Despite several studies investigating the impact of amino acids on zinc absorption, contradictory results have prevented the formulation of a general statement regarding the effect of amino acids on zinc bioavailability [10,31,32,33,34,35,36,37,38,39,40,41].

In this context, only a few studies have compared the bioavailability of various Zn supplements, likely due to the lengthy and laborious fecal collection required to calculate the mass balance between the administered isotopic dose and subsequent tracer excretion [42]. A dual-isotope method has been developed to estimate zinc absorption without prolonged fecal collection [43]; however, this method remains invasive, requiring an intravenous infusion and the purchase of two isotopes. A simpler and less expensive single-isotope method has also been developed [44], which has demonstrated similar results to the dual-isotope method for estimating Zn absorption in healthy adults with normal Zn status [44]. Zn concentrations in urine reflected those in plasma, while percentage absorption of ^67^Zn in urine was not affected by age, weight (which is proportional to plasma volume), gender, or Zn status [44]. This method was deemed less sensitive than the dual-isotope method [44]; however, its widespread potential application is significant because it is inexpensive, non-invasive, and straightforward to execute. Furthermore, it can provide comparative information when different interventions are evaluated within the same population.

In this preliminary study, we employed this method to determine whether differences in fractional Zn absorption (FZA) could be observed when Zn was administered in sulfate salt, gluconate, or aspartate form with milk to healthy human volunteers.

## 2. Materials and Methods

### 2.1. Subjects

Eight healthy adults (no recent episodes of gastroenteritis, no chronic gastrointestinal disorders, afebrile), four males and four females (age matched) with an age range of 25–50 years and with normal Zn status were recruited from Ancona, Italy. The research was approved by the Ethic Committee of Marche Region (prot. 34431/P; 08/07/2009) and all participants gave written, informed consent after the nature, purpose, inconveniences, risks, and benefits of participation were explained.

### 2.2. Study Design

The study was designed in two separate phases:

− The first phase aimed at evaluating the precision and accuracy of various isotopic ratios (^66^Zn/^70^Zn, ^64^Zn/^70^Zn and ^68^Zn/^70^Zn) in baseline urine samples, as well as comparing the kinetic dynamics of ^70^Zn isotopic enrichment (%E) in plasma and urine after oral administration of 2 mg ^70^ZnSO_4_. The results obtained in this phase were used to establish the best isotopic ratio used in the second phase.
− The second phase aimed at measuring the FZA 48 h after the oral ingestion of 2 mg of ^70^Zn given in the form of three different complexes: ^70^Zn-Aspartate (Zn-Asp), ^70^Zn-Sulfate hydrate (^70^ZnSO_4_), and ^70^Zn-Gluconate (Zn-Glu). This phase was carried out 6 months after the first phase to evaluate any seasonal differences that could affect the %E of ^70^ZnSO_4_.

### 2.3. Materials

Trace element-free sulfuric acid, sodium hydroxide (BDH Inc., Toronto, ON, Canada) and high-purity distilled deionized water filtered through a 0.22 mm Millipore filtration system (Millipore, Bedford, MA, USA) were used throughout the preparation of the oral doses of Zn isotopes. All laboratory work was completed in a clean laboratory under a laminar flow hood. Acid-washed glassware and plastics were used throughout. Isotopes of ^70^Zn were obtained as ^70^Zn-Asp ((C_4_H_6_NO_4_)_2_^70^Zn), ^70^ZnSO_4_, and ^70^Zn-Glu (C_12_H_22_O_14_Zn), all at 72% isotopic purity, (Sigma Aldrich, Custom Synthesis service, Milano, Italy).

The isotopes complexes were tested for sterility and pyrogenicity by the pharmacy of INRCA and encapsulated in gelatin. The oral capsules had a final total amount of 2.0 mg of ^70^Zn, with each one independently in the form of the complex. The oral doses were consumed in the morning with an individual milk feed.

### 2.4. Procedure

Phase 1. Baseline urine and plasma samples were obtained before the administration of the ^70^ZnSO_4_ capsule_._ An oral dose of 2.0 mg ^70^ZnSO_4_ was used as Zn supplement. This dose was previously shown to be adequate to achieve a urinary Zn absorption after 48 h to determine accurately the %E [43]. The subjects were instructed to take the Zn capsule in the morning while in a fasted state, with only one drink of milk (approximately 200 mL) allowed to aid ingestion. After the baseline sample collection, multiple urine and plasma samples were collected after 12, 24, 36, 48, 120, 168 and 196 h (±45 min) from the administration of the ^70^ZnSO_4_ capsule. The Zn isotope ratio was obtained for ^66^Zn/^70^Zn, ^64^Zn/^70^Zn, and ^68^Zn/^70^Zn in all samples and the Zn %E was calculated from the first follow up time (12 h) up to 196 h from the baseline.

Phase 2. Six months following Phase 1, baseline urine samples were collected from the same group of eight healthy adults who participated in Phase 1. Prior to receiving three different Zn complexes, participants were randomly allocated to three groups: two groups of three subjects and one group of two subjects. Each group underwent a rotating schedule of single oral administrations, including ^70^Zn-Glu (2 mg ^70^Zn), ^70^Zn-Asp (2 mg ^70^Zn), and ^70^ZnSO_4_ (2 mg ^70^Zn). Each group received all the single doses after a two-week wash-up period between each administration. The subjects were instructed to take the Zn capsule in the morning while in a fasted state, with only one drink of milk (approximately 200 mL) allowed to aid ingestion, as already performed in the first phase. The design of this study was a double-blind three-period crossover trial. Baseline and 48 h urine samples were collected after each period. FZA was calculated following the method (see Section 2.5) described by Yeung et al. [44].

#### 2.4.1. Zn Determination in Urine and Plasma Samples

Measurement of Zn in plasma and urine samples was performed with a Thermo XII Series ICP-MS (Thermo Electron Corporation, Waltham, MA, USA) following the manufacturer recommendations and as already performed in previous studies [45]. The instrument was operated in standard mode with a Peltier cooled impact bead spray chamber and a single-piece quartz torch (1.5 mm i.d. injector) together with Xi interface cones and a Cetac-ASX 100 autosampler (CETAC Technologies, Omaha, Nebraska). A Burgener Trace nebulizer was used because this device does not block during the aspiration of clinical samples. Urine and plasma samples were centrifuged for 10 min at 20,000× *g* and the supernatants were diluted 1:10 (total volume 1 mL) with a diluent containing 0.1% Triton X-100 (BDH Chemicals), 0.1% Trace Select Ultra HNO_3_ (Sigma-Aldrich), and 10 ppb Rh (Merck, Darmstadt, Germany) as internal standard. External multielement calibration solutions containing Zn (blank and 0.5–500 ppb) were prepared by serial dilution of a parent solution (Inorganic Ventures, Christiansburg, VA, USA) using the same diluent used for the samples. Sample uptake was optimized for 90 s at 14 rpm (about 0.4 mL/min) and the wash time was set at 60 s (wash solution was 1% Triton X-100 and 0.1% HNO_3_). Data (three repeats per sample) were acquired for ^66^Zn.

#### 2.4.2. Determination of Zn Isotope Ratios in Urine and Plasma Samples

Zn isotope ratios ^66^Zn/^70^Zn, ^68^Zn/^70^Zn, and ^64^Zn/^70^Zn were automatically calculated by the instrument software (Plasmalab v2.5.9, Thermo Fischer Scientific, Bremen, Germany). Zn isotopes were measured by adapting methods used for direct measurement of trace elements in human blood, serum, or plasma [12]. The obtained isotope ratios were corrected for mass bias by applying a linear model that used the following expression (Rr = Rm/(1 + KΔM), where Rr is the real isotopic ratio, Rm is the measured isotopic ratio, ΔM the mass difference between the isotopes, and K is the mass bias factor (in this case −0.02). Accuracy of the isotope ratio measurements was verified by analysis of a Zn nitrate standard (VWR Scientific, Radnor, PA, USA). 

### 2.5. Isotopic Zn Absorption

Fractional Absorption of Zn (FZA) was determined via the ‘single-isotope’ methodology [44] using the following equation:

FA = k (mg) × (%E of oral dose in urine/oral dose (mg)),

where k (mg) is the value of 1.60 mg previously calculated [13], oral dose is 2 mg and %E of oral dose in the urine is calculated as:

%E ^70^Zn = ((^70^Zn/^x^Zn)_enr_ − (^70^Zn/^x^Zn)_base_) × %NA^x^Zn,

where ^x^Zn refers to ^64^Zn, ^66^Zn, and ^68^Zn and the %NA^x^Zn is the natural percentage of isotopic abundance of Zn (i.e., 48.63% for ^64^Zn, 27.90% for ^66^Zn, and 18.75 for ^68^Zn).

### 2.6. Sample Size Detectable Difference 

The sample size calculation (*n* = 8) was performed with G*Power (version 3.1.5) based on an ANOVA for repeated measurement test analysis, assuming an acceptable difference in at least one isotopic complex to be equivalent to the standard deviation of %E, previously observed to be 37% [11]. Power was set at 0.95, while alpha was set at 0.05.

### 2.7. Statistical Analysis

Statistical analysis was performed by ANOVA for the repeated measures package included in SPSS v 16. Least significant difference without adjustments was used for multiple comparison among the three experimental groups.

## 3. Results

### 3.1. Basal Plasma Zn and Isotope Ratios 

Basal plasma Zn of the study population was equal to 12.74 ± 1.39 µM (min = 11.03 µM; max = 14.45 µM). None of the volunteers showed plasma Zn values below/above the reference ranges concerning sex and age [46]. The basal values of ^66^Zn/^70^Zn in urine were not significantly different from those in plasma, with mean values of 37.1 ± 2.5 and 37.0 ± 0.3, respectively, according to a paired *t*-test (*p* = 0.23).

The accuracy of the isotope ratio measurements was verified by the analysis of a Zn nitrate standard (VWR Scientific). The relative accuracy of ^66^Zn/^70^Zn, ^64^Zn/^70^Zn, and ^68^Zn/^70^Zn isotope ratios was within 1.65%, 5.65%, and 2.89%, respectively. The repeated baseline urine samples were used to determine the precision of the isotopic measurements at natural abundance. The relative precision (expressed as standard deviation %) was 2.67, 3.54, and 3.22, for ^66^Zn/^70^Zn, ^64^Zn/^70^Zn, and ^68^Zn/^70^Zn, respectively. 

Since the relative accuracy and precision of the isotope ratios calculated with ^66^Zn was better than those obtained with the ^68^Zn and ^64^Zn, we calculated and compared the %E by using ^66^Zn/^70^Zn. However, the results were not affected by changing the isotope used for the measurement and calculations (*p* > 0.05).

### 3.2. Kinetic of Isotopic Zn Enrichment

**Figure 1:**
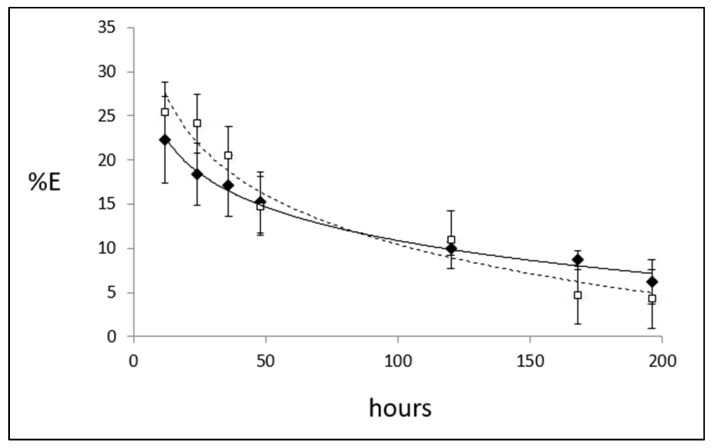
Kinetic of isotopic Zn enrichment in urine and plasma. This figure represents the kinetic from baseline up to 196 h of the Zn isotopic enrichment (%E) measured in plasma (white squares, dashed line) and urine (black squares, continuous line) samples after the administration of 2 mg of ^70^ZnSO_4_ from eight healthy volunteers. %E in the urine and plasma was calculated as: %E ^70^Zn = ((^70^Zn/^66^Zn)_enr_ − (^70^Zn/^66^Zn)_base_) × %NA^66^Zn, where %NA^66^Zn is the natural percentage of isotopic abundance of ^66^Zn (27.90%). No significant differences related to Zn %E were observed between urine and plasma samples.

The kinetic of the Zn %E up to 196 h was measured in the first phase of the study. After the administration of 2 mg of ^70^ZnSO_4_, a significant %E was observed in plasma samples after 12 h from baseline (Figure 1). Urine samples showed similar data to those of plasma (*p* > 0.05) at each time point (Figure 1). After 48 h, urine and plasma samples showed the most similar values (Figure 1), thus confirming the validity of the choice to measure Zn %E in urine after 48 h from baseline [44]. After 48 h from the administration, ^66^Zn/^70^Zn values in urine and plasma were equal to 32.0 ± 1.8 and 32.2 ± 1.2, respectively. No differences concerning ^66^Zn/^70^Zn were found between urine and plasma samples after 48 h from the administration (*p* = 0.16). 

The Zn %E did not display any difference (*p* = 0.688) between urine (13.7 ± 4.4) and plasma (14.7 ± 6.9) samples after 48 h from baseline. We also measured the % E in urine after 8 and 15 days from the initial administration. The mean value was found to be 6.20 ± 4.96 after 8 days and continued to diminish; after 15 days, the % E was indistinguishable from baseline (−0.75 ± 10.82).

### 3.3. Isotopic Zn Absorption of Different Zn Complexes in Healthy Human Volunteers

About 6 months after the end of the first administration, a second administration of ZnSO_4_ was provided. No significant difference between the two seasonal measures of the Zn % E in urine after 48 h from baseline was detected (*p* = 0.425). 

**Figure 2:**
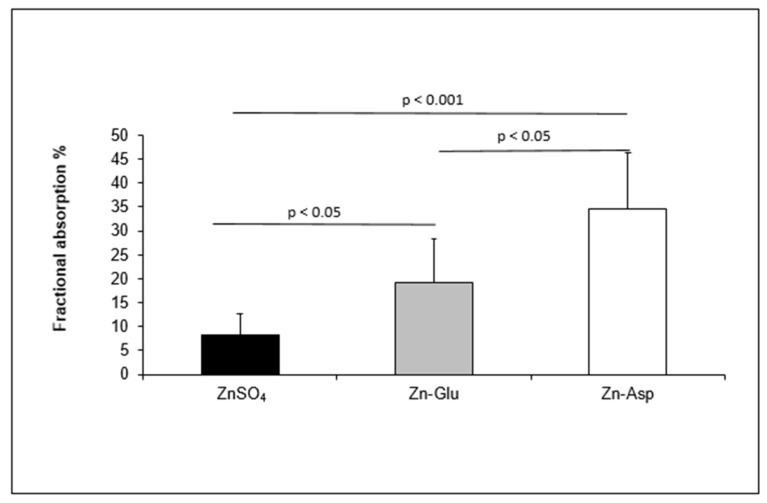
Comparison between the fractional Zn absorption obtained by oral administration of three different Zn complexes. The figure represents the comparison between the FZA measured after 48 h of oral administration of ZnSO_4_, Zn-Glu, and Zn-Asp in eight healthy human volunteers. FZA was determined via the ‘single-isotope’ methodology [44] using the following equation: FA = k (mg) × (%E of oral dose in urine/oral dose (mg)), where k (mg) is the value of 1.60 mg previously calculated [13], oral dose is 2 mg, and %E of oral dose in the urine is calculated as: %E ^70^Zn = ((^70^Zn/^66^Zn)_enr_ − (^70^Zn/^66^Zn)_base_) × %NA^66^Zn, where the %NA^66^Zn is the natural percentage of isotopic abundance of ^66^Zn (27.90%). ZnSO_4_: zinc-sulphate; Zn-Glu: zinc-gluconate; Zn-Asp: Zn-Aspartate; %E: percentage enrichment.

Table 1 shows a significantly different FZA between Zn complexes. Sex did not affect the differences observed between complexes (Table 1). The highest FZA was observed after consumption of Zn-Asp (mean ± SE = 34.58 ± 3.58), while the lowest was observed after consumption of ZnSO_4_ (mean ± SE = 8.94 ± 1.19). FZA in the form of Zn-Glu (mean ± SE = 19.13 ± 3.53) was higher than ZnSO_4_ but lower than Zn-Asp. Comparison and significance data are reported in Figure 2.

## 4. Discussion

This preliminary study aimed at evaluating the most efficient Zn-complex among Zn-Asp, ZnSO_4_, and Zn-Glu to improve Zn absorption with milk ingestion. 

At our knowledge, the data herein presented provide the first experimental evidence that Zn administered in the form of Zn-Asp can enrich Zn human content more efficiently than other forms of Zn supplements, such as Zn-Glu and ZnSO_4_. In particular, Zn-Asp increased Zn absorption more than 2-fold more than ZnSO_4_ and about 1.5-fold more than Zn-Glu. These data are in agreement with previous observations by which Zn levels in the prostate gland and testis tissues were higher in rats treated with Zn-Asp when compared to treatment with ZnSO_4_ [47]. Hence, the combination of Zn with the amino acid aspartate appears to be an efficient formula for preparation of Zn supplements. 

To date, it has not been possible to provide general statements on the effect of amino acids on Zn bioavailability due to the existence of many contradictory studies [33,35,36,37,38,39,40]. However, several mechanisms might explain the improved absorption of Zn-Asp compared to Zn-Glu and ZnSO_4_ observed in this study.

At first, amino acids complexes with Zn can keep the cation in solution, thus increasing its bioavailability in the intestinal lumen [35,48]. Alternatively, they might favor its absorption via amino acid transporters [34,35].

For example, studies on cells or perfused intestines have shown a positive effect of amino acids, such as histidine, on Zn uptake [37,48]. However, it should be noted that this is not true for all amino acids, likely due to differences in their binding affinity with Zn [48]. For instance, methionine, which has a weak binding affinity with Zn, may not have a significant effect on Zn uptake [36,48]. Hence, the stable binding of Zn with aspartate may explain why the Zn-Asp complex used in this study provided a higher absorption than ZnSO_4_ which, in turn, can also form insoluble complexes with other dietary components, such as phytates [48].

In this study, the subjects consumed the Zn supplements in the form of capsules ingested with the help of a milk drink. Milk can potentially affect the Zn absorption [14] due to the presence of certain compounds (i.e., caseins) that can bind to Zn and form complexes that are less readily absorbed in the small intestine [48]. Moreover, the high amount of calcium in milk might have affected Zn absorption; however, several studies have provided evidence that the addition of a large amount of calcium to a single meal does not affect Zn absorption [49,50].

Although this manuscript did not investigate the possible mechanisms that underlie Zn absorption, the data herein presented could form the rationale to support intervention trials with Zn-Asp in populations where Zn absorption could be limited, such as older adults. Indeed, there is already evidence that a moderate supplementation of Zn-Asp (10 mg Zn++/day × 30 days) in an elderly population (selected on the basis of plasma Zn deficiency) is safe and can positively modulate immune, lipid, antioxidant, and psychological parameters [31,51]. In another study, 10 mg/day of Zn-Asp was able to induce regulatory T cells, resulting in suppression of allogeneic immunity, which is characterized by chronic cell-dominated immune reaction [52]. Moreover, a preliminary trial with Zn-Glu-fortified milk was recently proven to be safe and provide potential health benefits in very old people (above 85 years) [41].

The method used to evaluate the FZA was robust and reproducible. Indeed, after 6 months the isotopic Zn %E of ZnSO_4_ did not show any significant difference. Moreover, the estimation of FZA through the monitoring of urine Zn excretion after 48 h confirmed it to be an accurate and non-invasive tool [43]. Considering that any intervention such as a blood withdrawal in older adults could determine several problems, the confirmation of urine samples as a powerful matrix to monitor Zn absorption is not negligible. Finally, the precision <3% obtained in our study suggests that ^66^Zn/^70^Zn is the most accurate isotopic Zn measure within a body fluid.

The results presented here also suggest that 2 mg of Zn given as Zn-Asp in milk was safe and improved FZA for a limited time period. These data do not take into account a possible effect of the amino acids on the Zn excretion, which could confound the results. However, only Histidine was reported to affect the excretion of Zn in the urine [48] and, to our knowledge, there is no report showing increased excretion of Zn after administration of Asparagine or Glutamic acid.

### Limitations of the Study

Having used the single-isotope method to estimate data of isotopic Zn absorption [44], the data herein presented should be considered valid only for the comparative purpose of this study and cannot be claimed as absolute values of %E. Indeed, this would have required the inopportune assumption that the k value calculated in the original paper on the single-isotope method was valid for the individuals sampled here, while it is known that this factor presents strong inter-individual variability [53]. Moreover, the resulting %E determinations can also be affected by the time (48 h) proposed to sample urine in the single-isotope method, rather than the conventional (60 h) time used by current practice users of the validated dual-isotope method. Nevertheless, the adoption of a crossover design ensures that the comparison of the %E among the three forms of Zn is similarly biased, thus allowing comparison among the groups for this preliminary study. We did not find significant differences between urine and plasma isotope ratios after 48 h of administration of the isotopes; thus, at least in our volunteers, the urine isotope ratios at 48 h could be considered a good estimation of plasma isotope composition. We also found that the signal after this period was shown to be relatively high compared to later sampling, thus ensuring that contaminations with residual absorption from the previous dose due to the crossover design were negligible.

## 5. Conclusions

In conclusion, Zn is an essential trace element that plays numerous essential roles in the human body. Zn deficiency can lead to various health problems and adequate Zn intake is crucial for maintaining optimal health. Zn supplementation can be an effective way to treat Zn deficiency and improve health outcomes; however, it should be used with caution and under the guidance of a healthcare professional. 

The results from this study might be useful as a rationale for conducting further Zn supplementation studies with Zn-Asp, especially in populations where absorption might be impaired, such as older adults.

## Figures and Tables

**Table 1:** ANOVA test of Zn-complex effect on fractional Zn absorption.

Source	Type III Sum of Squares	F	Sig. *
Zn-Complex ^a^	2665.88	17.33	<0.001
Zn-Complex × Sex	365.45	2.38	0.135

* p-value related to the association between isotopic FZA within three Zn complexes with sex interaction. ^a^ Zn Complexes include ZnSO_4_, Zn-Glu, and Zn-Asp.

## Data Availability

Data are available at request.
